# How do UK general practice staff understand and manage prediabetes? A focus group study

**DOI:** 10.3399/BJGPO.2021.0166

**Published:** 2022-05-18

**Authors:** Eleanor Barry, Trisha Greenhalgh

**Affiliations:** 1 Department of Primary Care Health Sciences, University Department in Oxford, Oxford, UK

**Keywords:** Prevention, Qualitative research, Diabetes

## Abstract

**Background:**

Preventing type 2 diabetes is a national priority; one aspect is the identification and active management of ‘prediabetes’ through lifestyle change.

**Aim:**

To explore what primary care clinicians understood by ‘prediabetes’, how they communicated this diagnosis to people, how they delivered lifestyle advice, and their views on barriers to lifestyle change.

**Design & setting:**

Three focus groups were undertaken with 25 individuals from primary care teams (GPs, nurses, and healthcare assistants) in Newham, a deprived and ethnically diverse part of London, UK.

**Method:**

Recordings were transcribed verbatim and analysed thematically before integrating social and behavioural science theories.

**Results:**

Focus groups participants described four main influences on their management of prediabetes in the consultation: social determinants, clinical aspects of diagnosis and management, patient motivation and behaviour change, and long-term care. Since most felt unable to address social determinants such as poverty, discussions with patients tended to focus on attempts to change individual behaviours and achieve particular numerical targets, with limited attention to the social context in which behaviours would play out.

**Conclusion:**

Type two diabetes prevention efforts in general practice may fail to address the upstream causes of this disease. A narrow focus on numerical targets and decontextualised behaviours overlooks the social complexity of human behaviour and lifestyle choices. Within the consultation, the authors recommend that greater attention is paid to discussing the social context and meaning of particular behaviours. Beyond the consultation, collaboration between primary care clinicians, public health bodies, and local governments is required to address community-level constraints to behaviour change.

## How this fits in

People with prediabetes are typically managed with lifestyle advice in general practice. There is limited research on how clinicians inform people of the diagnosis of prediabetes and deliver lifestyle advice. This focus group study found that advice was often narrowly focused on individual behaviour change and numerical targets for glycaemic biomarkers. Clinicians recognised the social determinants that influenced a patient’s risk of developing diabetes, but felt powerless to address these. Individual consultations for prediabetes should be supplemented by community-level action on social determinants

## Introduction

One-third of people who died in the first wave of the COVID-19 pandemic had diabetes.^1^ Risk factors for type two diabetes (ethnicity, deprivation, and obesity) were also associated with poorer outcomes from COVID-19.^2–4^ Preventing type two diabetes was a national priority before the pandemic,^5–7^ and as general practice resumes chronic disease management clinics, it has become one again.^8^

National Institute for Health and Care Excellence (NICE) Guideline PH38^7^ states that those with an elevated HbA1c (42–47 mmol/mol) or impaired fasting glucose (5.7–6.9 mmol/L or WHO cut offs 6.1–6.9 mmol/L) are diagnosed with ‘non-diabetic hyperglycaemia’, also known as ‘prediabetes’. People with non-diabetic hyperglycaemia should be reviewed annually for disease progression and considered for prevention programmes. The NHS Diabetes Prevention Programme (NHS DPP) is now available nationwide, but completion is limited (19% of participants completing six out of 13 sessions) with high attrition rates in deprived areas and participants of Black or Asian ethnicity.^9,10^ General practice plays a central role in the diabetes prevention pathway. Clinicians make decisions on testing and diagnosis, communicate results, and give lifestyle advice before offering referrals to prevention programmes. General practice provides continuity of care, annual reviews of people at risk, and opportunistic delivery of health promotion messages.

How practice teams communicate a prediabetes diagnosis and deliver subsequent health promotion message affects how people respond and influences lifestyle change.^11,12^ Prediabetes may have different meanings, interpretations and implications for clinicians and patients, with a new risk identity for those given this diagnosis.^13^ The literature has a limited focus the practice team’s perspectives on this condition.^14^

Given the central role of general practice in diabetes prevention, the authors undertook focus groups with practice staff (pre-COVID-19) to understand their views and perspectives on the prediabetes diagnosis, supporting behaviour change, and delivering lifestyle messages.

### Objectives

The objective was to explore, from the accounts of practice staff:

1. How they convey a diagnosis of prediabetes (or NDHG) to patients.
2. How they encourage and support behaviour change in such patients with a view to preventing progression to type two diabetes.
3. How they view the barriers and enablers to lifestyle change.

## Method

### Study context and governance

An interpretivist approach was taken, seeking to explore how prevention strategies are socially and culturally shaped while investigating the complexities that underpin health-related behaviours. The authors acknowledge that the study’s findings will have been influenced by their professional roles (as GPs), prior assumptions, and the context in which data is gathered and analysed.^15,16^

The study was a component of a doctoral research study funded by an NIHR Doctoral Research Fellowship. A study protocol and all study materials (consent forms, information sheets, interview topic summaries) were approved by the University of Oxford who sponsored the study and by an NHS REC (London-Surrey REC 12^th^ April 2018) and HRA. Data were managed in accordance with the data protection policies of the University of Oxford. EQUATOR reporting guidelines were followed in the reporting of this study.^16^

### Study setting

Newham, an east London borough, is a deprived area with 37% of residents living in poverty.^17^ It is an ethnically diverse community, with 45% of the population identifying as Asian ethnicity, 26.5% as White, and 17.8% as Black.^18^ Newham has a high diabetes prevalence due to the interplay of social, environmental, and economic influences.^6^ The Clinical Commissioning Group (CCG) commissions practices to maintain a register of people with prediabetes and incentivises them to undertake patient annual reviews, checking for diabetes, reviewing cardiovascular risk factors, and delivering health promotion messages.

### Sampling

Practices were recruited from three sites in Newham. Two practices were purposively selected because of their differing population demographics. The third practice responded to an NIHR Clinical Research Network email invitation to join the study. The practices serve populations with different ethnic makeups, giving a variety of perspectives. Newham is not statistically representative of a UK population, but themes and concepts developed from the analysis may resonate with teams in deprived areas with a high diabetes prevalence.

### Focus groups

Three focus groups were undertaken with doctors, nurses, health care assistants, clinical pharmacists, and managers in 2018. Open questions allowed participants to express opinions and generate discussions within the groups. A topic guide (reproduced in Box 1), guided exploration of their interpretation of prediabetes; how they said they communicated the diagnosis to patients; whether the diagnosis prompted behaviour change; how they supported patients in lifestyle change; and what the barriers to change were. Three fictional personas (case vignettes) were shared in each focus group to generate discussion on how clinicians might approach a specific case. Field notes describing the environment, non-verbal communication, and group interactions added contextual information to the analysis.

### Data analysis

Audio recordings were anonymised and transcribed verbatim. Each focus group transcript was read several times, with notes, thoughts, and initial codes handwritten in the margin. The data was further organised and managed using NVIVO (version 12) software. This allowed data to be organised and rearranged while maintaining its original source. NVIVO sub-codes were assigned beneath the initial indexing. The authors undertook a preliminary thematic analysis of the data,^19^ assigning broad codes to the data starting with the first focus group. Data from the other two focus groups was tested against the coding frame working in an iterative process, allowing for progressive refinement. Following this, the authors tested behavioural and social science theories against their analysis. A spreadsheet documented how coding decisions were made and how each theme was constructed.

### Theoretical approach

The authors align with critical social scientists and public health scholars such as Lupton, Baum, and Nettleton, who view the diagnoses of states such as prediabetes as a social construction.^20–23^ While numerical criteria are used to make the diagnosis, this categorisation tends to depict the cause of a disease as a failing of the individual’s biology. Delivering the diagnosis in a medical setting implies that the individual is now on a trajectory to disease development, but will be able to control that progression through behaviour change. This individualist discourse of risk diminishes the condition’s complexity and the role of the wider determinants of health in diabetes development.^23,24^ These authors depict this type of health promotion as a form of medical surveillance.^20,21^ Non-adherence to behaviour change with deviations outside defined ‘normal ranges’ may lead to victim-blaming, influencing how health messages are internalised by the individual.^23,25^ Focusing on the individual downplays, intentionally or not, the role of social, economic, and political influences in disease development, and draws attention away from upstream disease prevention models.^23,26^

## Results

### Description of sample and dataset

The focus groups included a wide range of practice staff, although GPs were predominant. Table 1 shows the demographic and professional characteristics of participants. The participants in each focus group had existing working relationships with one another, allowing the researcher to witness collaboration on a clinical scenario. These groups provided insight into the dynamics of the practice teams by how people interacted to answer questions, contradicted each other, built on ideas, and debated and responded to each other.^19,27^ The facilitator encouraged all members of the group to voice their opinions and respond to questions. While the group was allowed to explore ideas, a topic guide was used to keep the discussions relevant to prediabetes. Fictitious personas were used to see how the group approached clinical scenarios (see Supplementary Box S2 and Supplementary Figures S1-S3 online).

**Table 1.** Demographic and professional characteristics of study participants

Focus group number, participants (*n*)	Practice roles, *n*							
	GP	Practice nurse	HCA	Community pharmacist	Practice manager	GP trainee	Ethnic group, *n*	Sex, *n*
1 (7)	5	1	0	0	0	1	White = 3Asian = 2Afro-Carib = 2	M = 3F = 4
2 (11)	5	1	1	2	1	1	Afro-Carib = 6White = 2Asian = 3	M = 3F = 8
3 (7)	3	1	1	0	2	0	Asian = 6White = 1	M = 4F = 3
Total (25)	13	3	2	2	3	2	White = 6Afro-Carib = 8Asian = 11	M = 10F = 15

Afro-Carib = Afro-Caribbean. F = female. HCA = healthcare assistant. M = male.

### Theme 1: Diagnosing and managing prediabetes

#### Diagnosis

GPs understood the diagnosis of prediabetes as either a pathophysiological process or as an epidemiological condition defined by biomarker diagnostic thresholds.

*‘Chemically, it’s defined by decreased insulin sensitivity. And it’s a stage that happens gradually … towards the person then becoming diabetic.’* (FG1 GP)

Participants found numerical cut-offs useful in explaining to the patient that they were at risk. They conveyed the diagnosis in relation to a progressive linear trajectory to type two diabetes, emphasising that ‘prediabetes’ means ‘before diabetes’. Many depicted prediabetes as a definitive medical category that needed to be acted upon to prevent progression. The diagnosis was seen positively by most participants as a window of opportunity for action. It meant the patient could be ‘treated’ with lifestyle measures preventing disease progression:

*’you're identifying an intervention group, which changing something will have a positive outcome. Otherwise you wouldn't really have that range as prediabetic range, if it didn't have any clinical significance.’* (FG2 GP)

A minority of clinicians felt that the numerical thresholds caused some issues around overdiagnosis and uncertainty. Cut-offs alone did not identify those at most risk, with a high proportion of patients having an abnormal HbA1c. Additionally, people who had normal test results yet multiple risk factors for developing type two diabetes could be falsely reassured.

#### Monitoring and surveillance

The focus groups discussed the management of prediabetes as communicating the diagnosis, annual reviews, and surveillance and monitoring of the individual. In the annual reviews, the nurse or healthcare assistant monitors the patient’s weight, HbA1c, and blood pressure. Graphs of numerical results and colour scales were used to reflect back to the individual their progress and the implications of this. The clinicians found these tools helpful in delivering health promotion messages. Many participants appeared to assume that if the patient changes their behaviour then deterioration into diabetes will be stemmed:

*’Sometimes I used the colourful chart where the green is the safe numbers. Then the yellow, you know, the amber one is from 42 to 47. Then the red start from 48. So, when they come in, I just pull out the tape. And I say 'this is where you belonged before — now you’ve decided to jump into the yellow zone, and if you don't take care and you don't change your lifestyle by eating the right food, exercising and that, you jump into the red.’* (FG1 Nurse)

### Theme 2: Motivating patients to change their lifestyles

Participants placed the responsibility of reducing diabetes risk with the individual patient. People were asked to review their lifestyle choices and explore their ability to act, while having ‘discipline’ and ‘self-control’ to reduce their diabetes risk. This was done at diagnosis and at their annual reviews.

#### Fear of consequences

Fear of diabetes, medication, and diabetes complications were mentioned as a means to spur people into action. In some cases, participants indicated that they used scare tactics (depicting dire outcomes if no action was taken) to help motivate patients:

*‘And a lot of patients do not want to go on medication. I've encountered a lot that don't, ”Oh, I don't want blood pressure medication — don't give me that”, ”Then do something about your lifestyle, if you don't want medication”. Because that’s what we say for people with diabetes — they don't want it, but they need to do something to make a change, we can't just make the changes for you, you need to take control of your own life.‘* (FG3 Healthcare Assistant)

*’If they're scared about it, they're worried about it, then fine — I'll go with them, and we'll go with it. If they seem nonplussed, then my response is a lot more kind of aggressive, in the sense that, 'Do you not know what it means — you could lose your foot, you could lose your vision, you could have a heart attack'. Then you start putting the kind of complications in their mind, to get them worried about it.’* (FG1 GP)

Some clinicians considered that scare tactics were justifiable if they raised the awareness of risk and triggered the individual to act accordingly to change their lifestyles. Others recognised that these tactics had negative consequences, such as stigmatising people, which may prevent them from returning for review, or engaging in behaviour change or lifestyle interventions. This approach had to be tailored and timed correctly for each patient:

*’I think she got the fear of God in her, as soon we said, ”You're diabetic”.* [um] *Managed to change everything’* (FG1 GP)

*‘It was the first time I think anyone had actually addressed the fact that everything you're doing was adding up to him being a ticking time bomb, it was a wake-up call. For some people, they run away because they are scared. But other people, it will shock them into ”Okay, I've got to listen now”.’* (FG2 GP)

#### Theme 3: Long-term support

Clinical team members discussed the need for a long-term approach to supporting behaviour change based on patient-centred, relationship-orientated care. This approach is based on mutual trust between the clinician and patient, with knowledge of their social circumstances and cultural context. The focus group participants reported that this approach had a focus on small behaviour changes occurring incrementally over time. It required time for the patient to communicate their narrative, free from judgement, and feel that they have been heard. Encouraging the patient to decide what was possible, alongside showing an interest and offering kindness, was reported as key in this approach. Here the patients use their agency to decide what they can do, rather than being told what to do:

*’This is where it’s kind of important if the doctor gets it right and pitches it at the right level for the patient, and tries to understand the circumstances, rather than using circumstances against the patient.’* (FG1 GP)

*’I think concordance is another thing. To get the patient on board and feeling involved in the decision making about what changes they make. So, where he’s got practically everything wrong with him, you know? It might be useful to sort of put the ball in his court … And when they're made to feel like they're involved, and not just being told what to do, there’s a bigger chance of success with, with even small changes.’* (FG1 GP)

### Theme 4: Social determinants of health

Practice teams showed considerable depth and breadth of knowledge of the everyday lives of their patients. Every team recognised that people’s choices were constrained by poverty, cultural expectations, and the built environment. Understanding this context was vitally important, they felt, in tailoring health promotion messages. Table 2 gives examples of how these influences were discussed by the groups. Key barriers to behaviour change included financial insecurity, cultural norms, the obesogenic environment, gender roles, and health literacy. Housing and financial stresses were mentioned recurrently as structural barriers to behaviour change (Table 2, quote 1). Lifestyle change was seen as particularly difficult for women, (Table 2, quote 3): the demands of family life and work were prioritised above self-care. Overcrowded, poor quality housing has a large impact on preparing meals and exercising at home. The focus groups reported that people were constrained to eat what they can afford (quote 4, Table 2), which was often poor quality food that was high in sugar and salt. The high number of fast food outlets in the area reflects the demand for quick, cheap food and its cultural acceptance. Despite the availability of lifestyle interventions, like the NHS DPP, and the presence of green spaces, many participants felt that barriers to engagement in lifestyle were too overwhelming for patients:

**Table 2.** Quotes discussing the social determinants of health

Social determinant	Description
1. Financial insecurity	*Finance: ‘Yes. I mean, well what you are saying is if the person has a healthy mind. Now, he's unhealthy not because only of his not walking or walking. He's unhealthy also because he's occupied with something else, all the time. If like finances, worried about their finances, and things like that. The last thing they're going to be thinking about is their health. Going to be like ”I need to get these bills paid“. You know? So, I think their personal situations matter a lot, as well. And how they, you know, they deal with their lifestyle.‘* (FG3 GP)
2. Cultural norms about food and eating	*‘You know, will you — like changing the food, and things like that — they* [the family] *probably wouldn't like that. Like, “Why are you making it healthier, why are you changing our cooking method, we don't want it like it that”.' (FG3 Manager*)“*The other thing for me is oil in curries — I remember a patient who I was always telling to eat fish, and they brought me a little bowl full of oil, but a little piece of fish sitting in the oil. And so I try and say ”How much oil do you buy in a month?” And it's like five litres or something.’* (FG1 GP)
3. Gender roles and expectations	*‘The expectations of sometimes what men and women do. So, sometimes we think that you wouldn't get anywhere with men, but actually they're the ones who have the* [um] *permission within society to go to the gym, to be out, and not to have to do the housework and the childcare.* [um] *And for women, especially sort of women in their forties and fifties, that there's an expectation that you know, there is no social life, there's no* [um] *going out, you know, to go to an exercise class, and they're more at home.* [um] *And, and/or you need to ask permission to go out. And it's still — it surprises me that there's still quite a lot of that around.* (FG 2 GP)
4. Obesogenic environment	*‘FG1 GP: Just a walk round. You can see, you see all the chicken and chip shops. Fast food. Cheap and cheerful. So. Keep you happy for that moment, but after that, it depletes you, really. Drains you all the time.* *Interviewer: So do you think it's that there's not enough healthy options, or they're too expensive?* *FG1 GP: Yeah.* *Interviewer: Not enough?* *FG1 GP: There's not enough. Not enough* [healthy food options]. *And probably because it's too expensive.’*
5. Health literacy	*‘I guess sometimes the actual underlying knowledge about healthy eating isn't always there, as well.* [um] *Especially when you're dealing with someone who comes from a — is relatively isolated, who hasn't necessarily had the best schooling, is relatively new to the country. Changing everything round like eating, your entire life — you need to change everything, you need to eat what's on the NHS website — it's sometimes difficult to make that leap.’* (FG1 GP)

*’However, it’s easier said than done. I do know that I have not been able to control totally blood pressure just through lifestyle in any of my patients. You know? I've tried my best, because I think that if they reduce blood pressure, if they cut down on their smoking, and then the smoking has stopped, cut down on — they're helping … Unfortunately, I am still waiting for my first success. This has not happened.’* (FG3 GP)

## Discussion

### Summary

In this small focus group study undertaken in a deprived part of London, clinicians described four main influences on prediabetes and its management in the consultation: social determinants of health, clinical aspects of diagnosis and management, patient motivation and behaviour change, and long-term support. Since most felt unable to address social determinants such as poverty, discussions with patients tended to focus on attempts to change individual behaviours with limited attention to the social context in which behaviours would play out.

These findings suggest that clinicians in general practice are navigating two competing paradigms: individual and social. At the individual level, they are incentivised to subscribe to the biomedical model of disease prevention, which perpetuates an individualist focus encouraging people to take responsibility and control their behaviour to prevent diabetes. Prediabetes is portrayed as a medical certainty and people are given prescribed lifestyle advice (sometimes using controversial ‘scare tactics’)^28^ on how to prevent the perceived linear development to diabetes, and monitored using glycaemic biomarkers. Focusing on the individual foregrounds individual behaviours, and backgrounds the social causes of disease and underlying complexity of disease development.

At the societal level — as is often revealed through the ongoing therapeutic relationships possible in primary care — clinicians see first-hand how social determinants of health provide overwhelming structural barriers inhibiting patients’ behaviour change. This study’s findings suggest that they feel they have little choice but to give individualist health promotion messages, despite knowing that this is likely to have limited effect.

### Strengths and limitations

The focus groups worked well to gather large amounts of information from multiple perspectives, and to facilitate consensus-building in a short space of time.^27^ The focus groups allowed the authors to gather individual opinions and document group knowledge production.^19,29^ The groups generated insights and solutions to the mock scenarios that would not have occurred without the other participants.^23^

The study has limitations: all the practices took part in the local enhanced service and saw diabetes prevention as a priority. This may not be true for other areas, thus the views in these focus groups are not representative of all GPs. Furthermore, the small size of the study means the findings are preliminary. General practice has a hierarchical structure with GP partners the main employers, and this may have shaped how people answered questions and gave their opinions in the group discussions. GPs predominated in the focus groups: they communicated the diagnosis, discussed the risk of diabetes, and gave initial health promotion messages, while other staff (less well represented in the focus groups) undertook the annual reviews. The authors did not observe consultations as part of this study, but are conducting an ethnographic study of people with prediabetes, exploring their experience of being diagnosed with the pre-condition; how they internalise this diagnosis; how the diagnosis influences health related behaviours; and the role of risk regulators in behaviour change.

### Comparison with existing literature

The authors used a critical social science lens to explore prediabetes from the perspectives of primary care teams, which included the framing of the diagnosis, the delivery of health promotion messages, and the barriers to lifestyle change.

It is worth considering these findings in relation to Rose’s prevention paradox, which states that if you make societal level changes to reduce everyone’s risk of disease by a small amount, this is likely to lead to larger reductions in the incidence of disease than if prevention is targeted to ‘high-risk’ individuals.^26^ Currently, high-risk disease prevention strategies predominate in health policy. This reflects a ‘neoliberal’ approach to policy in general, emphasising individual responsibility and self-maintenance with less reliance on the state.^28^ This stance does not align with the view that individual health is largely the product of structural forces in society.^24^ Rose,^26^ and more recently Marmot,^30^ have argued that without addressing societal influences, it is unlikely that behaviour change will be possible for those with overwhelming structural barriers. For example, the poorest 10% of the country would need to spend 75% of their disposable income to meet the NHS’s Eatwell guidelines.^30^ A whole-population approach to diabetes prevention is politically problematic as it involves regulating corporations that make profits from selling high fat and high sugar content food, and that are also adept at political lobbying to support their positions. Affordability of healthy food and the commercial environment were discussed in all of the focus groups as some of the biggest barriers to lifestyle change. Given the devastating effects of COVID-19 in those with obesity, with diabetes, and from deprived areas, there have been calls for greater regulation on the food industry with improved affordability of healthy food options.^31,32^

Twenty years ago, Wylie *et al* ^33^ published a study of 34 clinicians’ views on the identification, treatment and management of impaired glucose tolerance. They found that GPs were reluctant to engage with health promotion, which they viewed as ’paternalistic‘ and medicalising the social causes of disease. The present study showed a greater commitment of clinicians to engage in disease prevention, perhaps due to specific financial incentives and the growth of functionally differentiated practice teams. Burch *et al* undertook qualitative interviews with primary care staff exploring the diagnosis and management of prediabetes in older populations.^34^ Similarly to the present study, they found that a person-centred approach was key in diagnosing and managing patients with an elevated HbA1c.

Others have undertaken qualitative work with clinicians as an adjunct to interviews with people labelled with prediabetes. Three studies aligned with the present study’s findings that health promotion strategies deliver individualist messages, asking individuals to control their disease prevention and using the diagnosis as a motivational tool.^12,13,35^ Twohig *et al* identified a number of key community constraints individuals faced when trying to engage with behaviour change programmes, but didn’t consider these as intervention opportunities.^11^

The empirical literature from critical public health is also relevant. Baum *et al* undertook qualitative interviews with health workers who, like the clinicians in he present study’s sample, described how the social determinants influenced their patients’ ability to partake in behaviour change.^36^ Acting as advocates for addressing these determinants was considered at odds with being a public servant, with structural barriers and workload pressures preventing clinicians engaging in the policy process.

### Implications for practice and policy

From April 2021, GP practices in England have been incentivised through the NHSE GP Contract to maintain a register of people with non-diabetic hyperglycaemia and invite them for annual blood tests to check for progression to diabetes. The present study’s findings suggest that it may be beneficial to shift health messages away from quantitative markers to exploring the patient’s lived experience and what is possible within their social context. The findings also suggest that a longer-term approach was a key prevention strategy in assisting people with behaviour change. Disease prevention policies do not currently reflect the importance of patient-centred care, with the current model of annual reviews placing a greater emphasis on numerical targets than on therapeutic relationships.^37^

GP practices in England are now organised into primary care networks tasked with addressing population health priorities. The focus groups suggested that primary care workers have unique insights into their patients’ social contexts and the barriers they face in trying to undertake lifestyle change. These patient narratives may be useful in identifying community-level opportunities and constraints on health.^38,39^ Additionally, Clinical Commissioning Groups are transitioning to Integrated Care Systems (ICS). These ICSs are tasked with addressing the upstream influences on health and reducing health inequalities. Primary care teams working alongside public health bodies and local government, may be key in tackling the upstream influences on health as part of a multi-faceted, place-based disease prevention strategy.^40–42^

This small study of primary care teams has highlighted the efforts made by staff to support people diagnosed with prediabetes and the powerlessness of such staff to address structural barriers to diabetes prevention. The system of structured care oriented around numerical targets and a linear model of progression from prediabetes to type two diabetes lend themselves to the use of individual health promotion messages and a narrative of inevitable progression unless behaviour changes — an approach that overlooks the complexity of diabetes development. These findings also highlighted the potential for relationship-based care to explore barriers to lifestyle change within the context of the individual’s social sphere. Primary care teams have key insights, through their patients’ narratives, into how the social determinants directly lead to disease development. New ICSs and Primary Care Networks are tasked with taking a population health approach to address the social determinants of health, reduce health inequalities, and reduce the burden of disease.^42^ Individual narratives play an important role — along with the co-ordinated working of primary care teams, public health teams, and local government — in maximising the benefit of new opportunities to address the community constraints to behaviour change.

## Data

This is a qualitative study and so does not rely or relate to any datasets.
